# A Deep Learning-Based Vision System Combining Detection and Tracking for Fast On-Line Citrus Sorting

**DOI:** 10.3389/fpls.2021.622062

**Published:** 2021-02-11

**Authors:** Yaohui Chen, Xiaosong An, Shumin Gao, Shanjun Li, Hanwen Kang

**Affiliations:** ^1^College of Engineering, Huazhong Agricultural University, Wuhan, China; ^2^Key Laboratory of Agricultural Equipment in Mid-Lower Yangtze River, Ministry of Agriculture and Rural Affairs, Wuhan, China; ^3^Citrus Mechanization Research Base, Ministry of Agriculture and Rural Affairs, Wuhan, China; ^4^China Agriculture (Citrus) Research System, Wuhan, China; ^5^National R&D Center for Citrus Preservation, Wuhan, China; ^6^Department of Mechanical and Aerospace Engineering, College of Engineering, Monash University, Clayton, VIC, Australia

**Keywords:** defective citrus sorting, CNN-based detector, SORT-based tracker, deep learning, vision system

## Abstract

Defective citrus fruits are manually sorted at the moment, which is a time-consuming and cost-expensive process with unsatisfactory accuracy. In this paper, we introduce a deep learning-based vision system implemented on a citrus processing line for fast on-line sorting. For the citrus fruits rotating randomly on the conveyor, a convolutional neural network-based detector was developed to detect and temporarily classify the defective ones, and a SORT algorithm-based tracker was adopted to record the classification information along their paths. The true categories of the citrus fruits were identified through the tracked historical information, resulting in high detection precision of 93.6%. Moreover, the linear Kalman filter model was applied to predict the future path of the fruits, which can be used to guide the robot arms to pick out the defective ones. Ultimately, this research presents a practical solution to realize on-line citrus sorting featuring low costs, high efficiency, and accuracy.

## 1. Introduction

Citrus is an important agricultural commodity produced in 140 countries, with the annual worldwide production estimated at over 110 million tons in the period 2016–2017 (Nazirul et al., 2017). For the fresh citrus fruit market, consumers demand fruits at a reasonable price without defects and diseases, which can be guaranteed by proper monitoring in the field and post-harvest quality inspection (Campbell et al., 2004). Traditionally, citrus fruits are manually sorted based on their external appearance in the packinghouse, which is time-consuming and cost-expensive. As the skill of the sorter varies from person to person, it is also an inaccurate process (Satpute and MJagdale, 2016). Therefore, it is necessary to develop automated systems to more effectively, economically, and accurately sort citrus fruits before they are sold in the market.

Damage to the citrus fruits can be caused by various issues, including insects in the field, bad practice in harvesting, infection penetration through injuries, or evolution of previous diseases during post-harvest storage (Holmes and Eckert, 1999; Burks et al., 2005). These diverse types of defects generate very different symptoms on their external appearance, making it challenging to develop non-destructive sorting methods with both high accuracy and efficiency. Hyperspectral image (HSI) technology, which inherits the advantages of both spectral and image analysis, has been adopted in several automated systems to detect the defects of agricultural products (Xing et al., 2004; Lee et al., 2014). However, applying HSI in real-time is difficult due to the relatively long time needed to acquire and analyze high-dimensional hyperspectral images. Multispectral image (MSI) technique captures images at only several specific wavelengths for higher efficiency and has been integrated into a real-time citrus sorting system (Qin et al., 2012). Despite a high accuracy of 95.3% achieved, it remains narrow as it focuses purely on citrus canker and new pests and diseases are still appearing. Traditional machine vision based on RGB cameras is a promising solution for on-line fruit sorting due to its high speed and low costs. This method has been adopted to investigate defective apples in a recent study with an average recognition accuracy of 90.2% (Zhang et al., 2017), but the accuracy is actually dependent on the features selected such as color, morphological and textural characteristics. The application of the NIR camera and NIR coded structured light, which aims to provide even lightness over the fruit surface, also complicates the system and increases the costs of postharvest handling.

In recent years, deep learning has become state of the art due to its strong adaptability to variances within the working scene, showing potentials for a variety of tasks within machine vision such as image classification (He et al., 2016), object detection (Redmon and Farhadi, 2017), and image segmentation (Kang et al., 2020). As it is capable of automatically learning the image features, better recognition accuracy can be expected compared with traditional image processing methods (Kang and Chen, 2020a). It has found its applications in various detection tasks in agriculture such as the pesticide residues of apples (Jiang et al., 2019), classes of garlic bulbs (Quoc et al., 2020), defects in cucumber (Liu Z. et al., 2018) and peaches (Sun et al., 2019), plant diseases (Picon et al., 2019), and automated robot harvesting (Kang and Chen, 2019, 2020a). In a more recent study, a deep learning-based vision sensor is developed to perform on-line detection of defective apples (Fan et al., 2020). However, since the apples are placed one by one on the conveyor for the simplicity of recognition, the speed of 5 apples per second is low and unsatisfactory for commercial production. As a result, none of the existing automated sorting systems is capable of achieving a good combination of high accuracy, efficiency, and low costs.

In this paper, we aim to develop a vision system based on deep learning, which can be implemented directly on a citrus processing line and perform fast on-line citrus sorting. To this end, a camera was mounted above the conveyor that transported multiple citrus fruits and presented their different surfaces during rotation. A novel detection-from-tracking sorting strategy was proposed that combined a detector and a tracker. The detector detected the defective surfaces of the fruits while the tracker memorized their classification information and tracked the location along their paths, and their true categories were identified through the historical information. The future paths of the defective fruits were also predicted using the Kalman filter algorithm, which can be adopted to control the robot arms to pick them out in real-time in future work.

## 2. Materials and Methods

### 2.1. System Configuration

#### 2.1.1. Samples

Sample oranges were harvested in August 2020 from a commercial orchard in Zigui, Yichang, China. This type of oranges is characterized by moderate sugar-to-acid ratio and varietal green to orange skin colors at the mature stage. A day after harvest the fruits with normal surface and several types of common defects were packed in cardboard boxes and sent to Wuhan, China via air flight.

**Figure 1:**
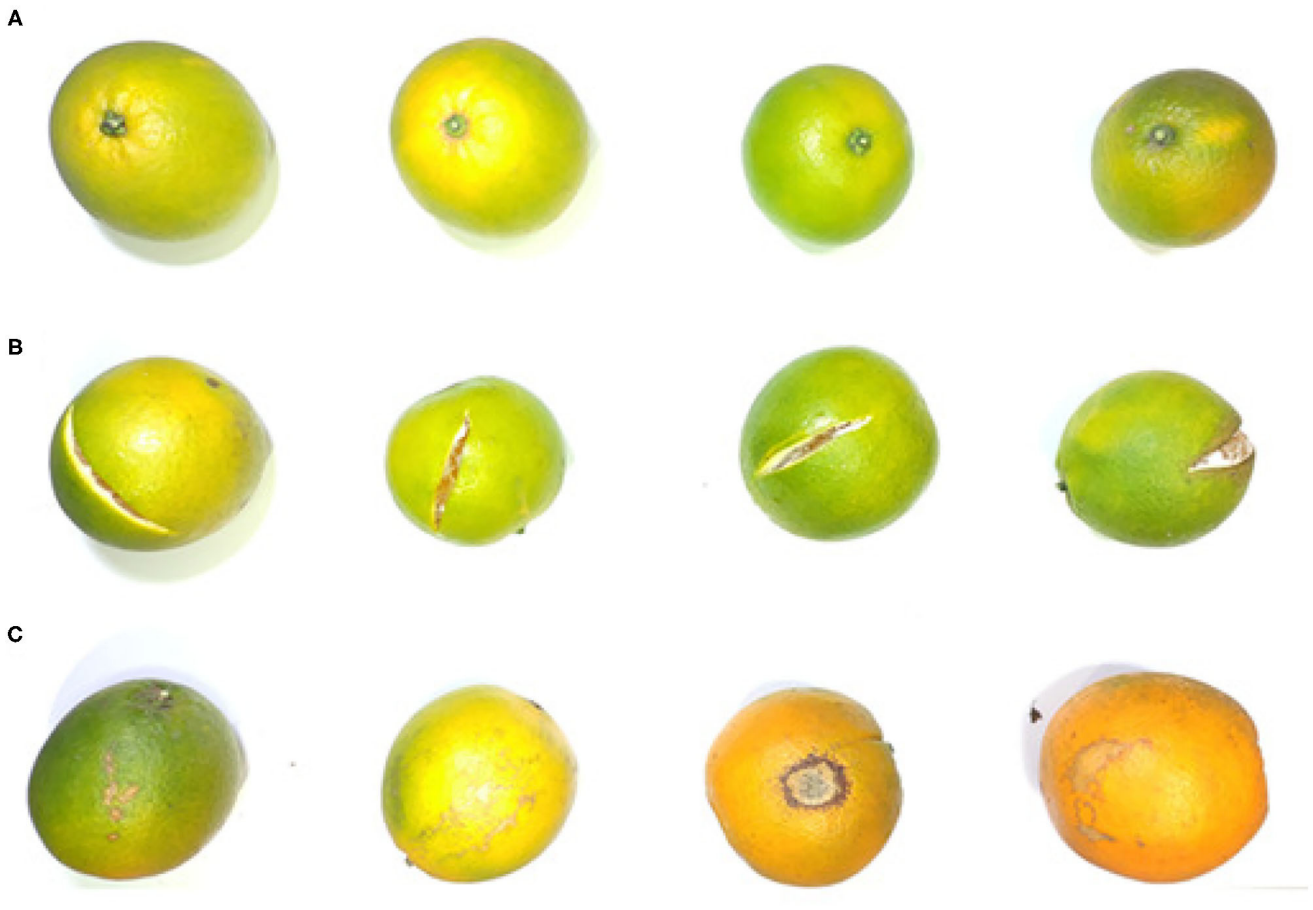
Sample oranges are classified into three categories, including **(A)** normal, **(B)** mechanical damaged (MD), and **(C)** with skin lesions (SL).

The oranges were first manually inspected and classified into three categories, including Normal (N), Mechanical damaged (MD), and with Skin Lesions (SL). Category N related to the oranges without any defects and ready for the fresh fruit market, as shown in Figure 1A. Category MD usually refers to those mechanically damaged by improper handling during the harvest or post-harvest process, while in this study it was defined as those with observable mechanical wounds and no other skin disorders for the simplicity of recognition, as shown in Figure 1B. For the fruits infected by fungi, pets or insects, the contrast between the sound peel and defects exists, and they were classified into Category SL, as shown in Figure 1C. A total of 300 oranges were randomly selected for the tests, of which 100 were from Category N, 100 from Category MD, and 100 from Category SL.

#### 2.1.2. Platform Setup and Vision System

**Figure 2:**
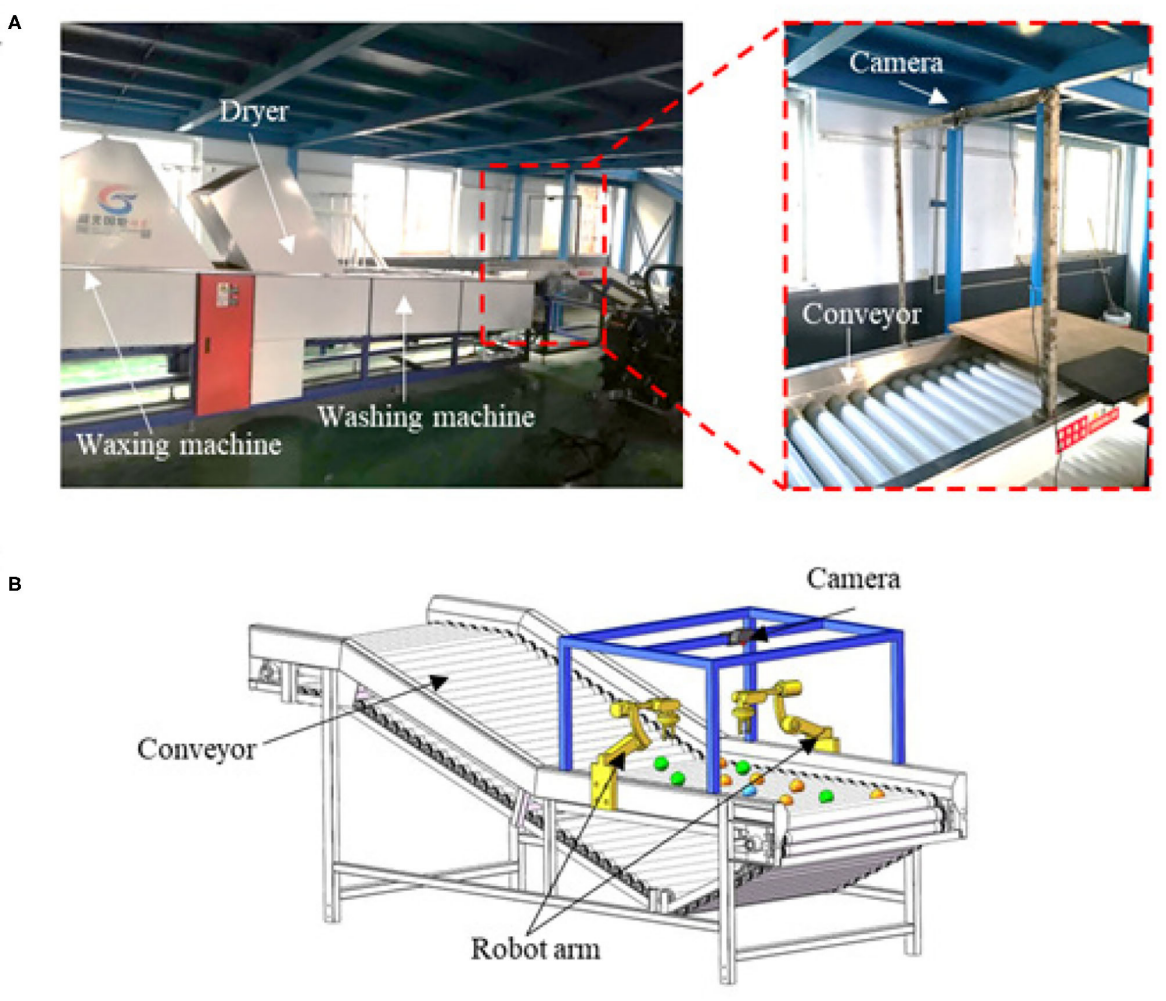
Platform setup and computer vision system. **(A)** The citrus processing line assembled in the lab, with a webcam mounted above the conveyor. **(B)** The diagram showing an automated citrus sorting system using a camera and robot arms, and the robot arms will be implemented in future work.

A commercially available citrus fruit processing line (GJDLX-5) was assembled in the lab and employed for automatic fruit cleaning and waxing, as shown in Figure 2A. Traditionally, the conveyor is employed to rotate the fruits freely so that the whole surface of each fruit can be manually inspected by the sorters. After that, the fruits with a sound surface are transported to the washing machine and waxing machine for processing. To automate the sorting process, a low-cost webcam (Gucee HD98) with an image resolution of 640 × 480 in 30 frames per second (FPS) was used to detect and track the defective fruits. The camera was mounted 0.5 m above the conveyor, and a 100 W LED light was used to enhance and balance the lighting conditions within the working space.

The vision-guided sorting process included two-steps: defective citrus detection and tracking. In the first step, the conveyor continuously rotated the oranges, letting the webcam view different surfaces of the oranges and detect the defective ones. A one-stage neural network-based detector Mobile-Citrus was therefore developed to detect and temporarily classify the citrus fruits into corresponding categories. In the second step, a tracker adopting a custom Simple Online and Real-time Tracking (SORT) algorithm was used to track the defective oranges (including Categories MD and SL) and predict their possible paths. The true categories of the oranges were then identified through the stored historical information. The predicted paths will be sent to the central control system to guide the robotic arms to pick out the defective ones, as shown in Figure 2B, which will be implemented in our future work.

**Figure 3:**
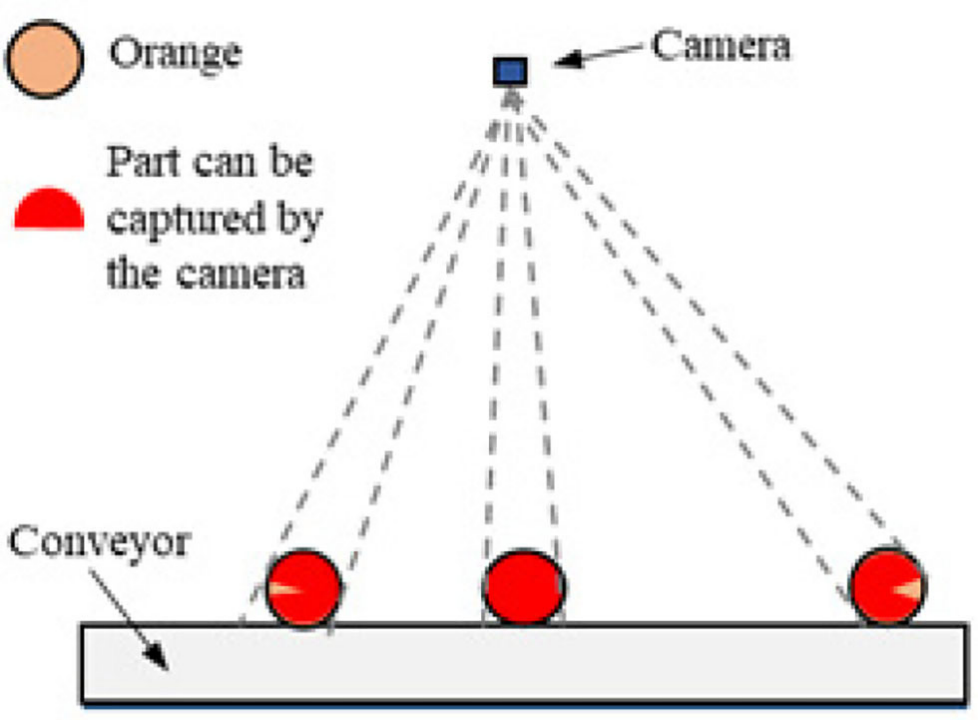
Diagram illustrating the part of the fruit surface captured by the camera in the rotation process.

As shown in Figure 3, although the camera could capture multiple images when the orange rotated, it might fail to observe the entire fruit surface of especially those near the edges of the conveyor. One solution is to implement multiple cameras to observe these oranges, which will be conducted in future work. During image acquisition in our experiment, we randomly picked 30–40 oranges from the 300 ones and placed them on the conveyor moving at a speed of 0.3 m/s for video taking each time. Forty videos at the frequency of 30 Hz were collected in total, and their duration were between 15 and 30 s. Among these videos, 30 were used for the developed detector. To avoid heavily overlapped information between neighboring frames, two frames per second were taken from each video sequence, resulting in 2400 images collected in total. Among these images, 700 were randomly selected to train the detector, another 500 as validation data, while the rest were used as test data. LabelImg tool was used to manually label the collected images in VOC format. The oranges were labeled as Category MD or SL only when the surfaces with damaged or lesional parts were captured. The remaining 10 videos were adopted to assess the performance of the combination of the detector and tracker. An object tracking dataset was also constructed to evaluate the proposed sorting strategy. This dataset included the bounding box and temporary category label of each orange presented in the video, with a specific number assigned to indicate its identity during the tracking process.

### 2.2. Defective Citrus Detection

#### 2.2.1. Network Model

**Figure 4:**
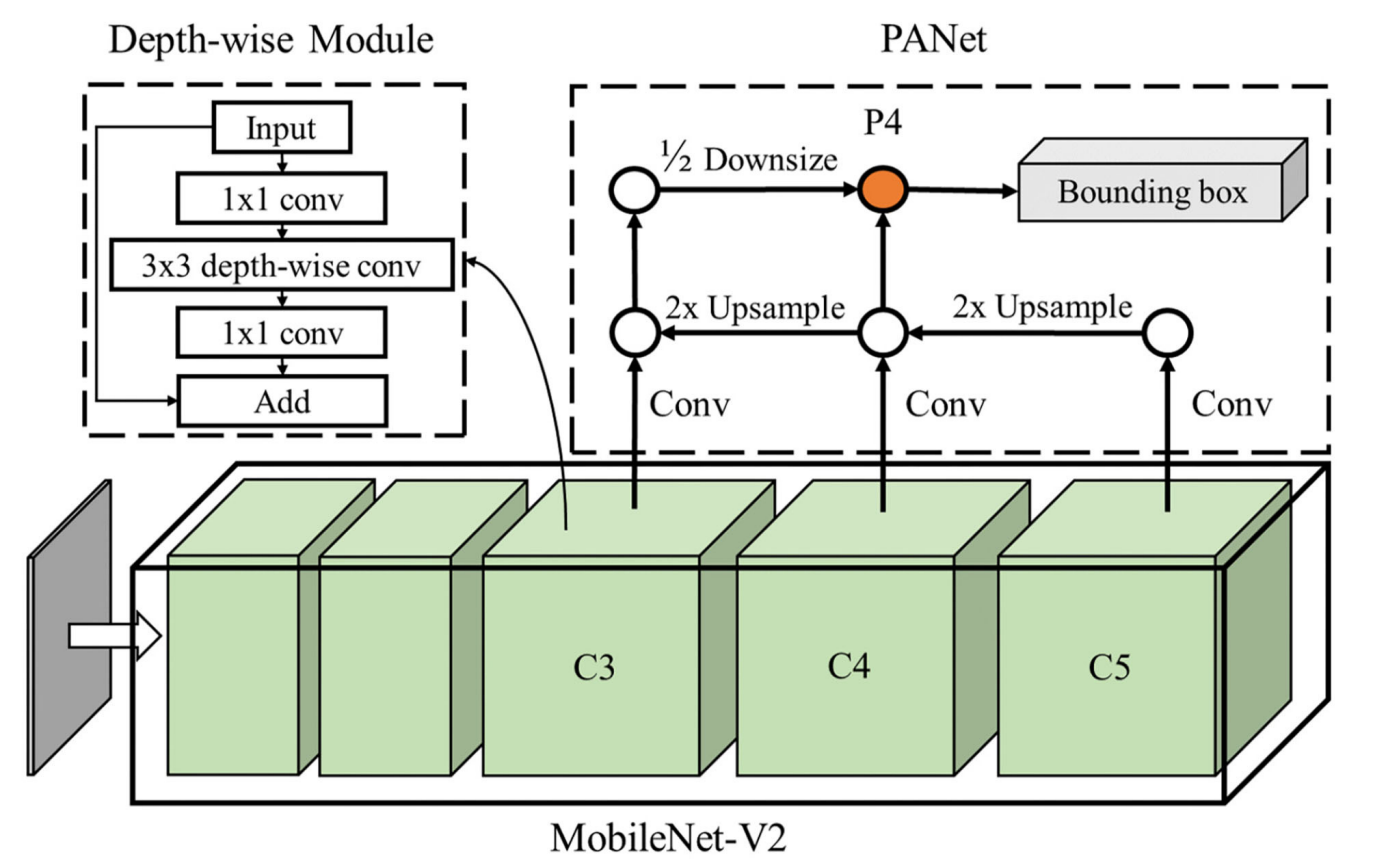
Network architecture of the detector, Mobile-Citrus, which includes MobileNet-V2 as the backbone and PANet as the detection branch. The output of the objects' bounding boxes are predicted from P4 level.

As convolution neural network (CNN)-based algorithms have shown superior performance in many computer vision tasks compared to traditional vision methods (Kang and Chen, 2019, 2020b), we developed a CNN-based detector Mobile-Citrus to detect the normal and defective oranges on the conveyor. CNN-based algorithms can be classified into two categories: two-stage detection networks with better performance in complex conditions and one-stage detection networks featuring better computational efficiency (Han et al., 2018). Since the proposed vision system should be capable of detecting defective oranges in a singular environment with real-time speed, a one-stage detection network was developed and applied in this work. The architecture of our proposed detection network included two parts: the network backbone and detection branch, as shown in Figure 4. Here, we applied a lightweight classification network MobileNet-V2 (Sandler et al., 2018) as the network backbone to extract multi-scale feature maps from the input images. After that, a Path-Aggregation Feature Pyramid Network (PANet) (Liu S. et al., 2018) was used to aggregate multiple-scale information from feature maps and detect the defective oranges.

#### 2.2.2. Network Backbone

The network backbone was used to extract and learn features and representations of the oranges within the input images. It adopted convolution layers to process the features of the oranges and pooling layers to aggregate the important features from the feature maps. As the pooling layers continuously shrunk the size of the images, the feature maps from the shallow levels comprised more spatial features of the oranges while the feature maps from the deep levels contained more semantic features. To improve the real-time computational performance, MobileNet-V2 using the depth-wise convolution operation was applied as the backbone due to its reduced computational complexity without sacrificing accuracy. Moreover, the shortcut design of the residual network module was introduced, which can largely improve the classification accuracy and training performance in deep networks. The proposed MobileNet-V2 included 18 depth-wise residual network modules in the model. The 8-times (C3), 16-times (C4), and 32-times (C5) size-reduced feature maps were used as the input for the detection branch to perform detection of the defective oranges.

#### 2.2.3. Detection Branch

Mobile-Citrus applied PANet to aggregate multiple-scale features from the backbone to perform the detection of defective citrus fruits. Compared to the standard Feature Pyramid Network (FPN), PANet introduced the top-down-top multiple-scale feature aggregation strategies for enhanced performance. As PANet can fuse both semantic features and spatial features to the corresponding detection head, it directly encoded the bounding box and classification information of the oranges in the tensors. The detection branch of Mobile-Citrus received C3, C4, and C5 feature maps from the backbone network, and the feature maps then followed the specific path of PANet and arrived at the detection head at C4 level. Since Mobile-Citrus was designed to sort oranges within a fixed scale, only the detection head at C4 level outputted the prediction of the bounding box and classification information of the defective oranges. The detection head of Mobile-Citrus followed the design of the YOLO network which includes the information of the confidence score, bounding box, and classification information within the tensors.

#### 2.2.4. Network Training

**Figure 5:**
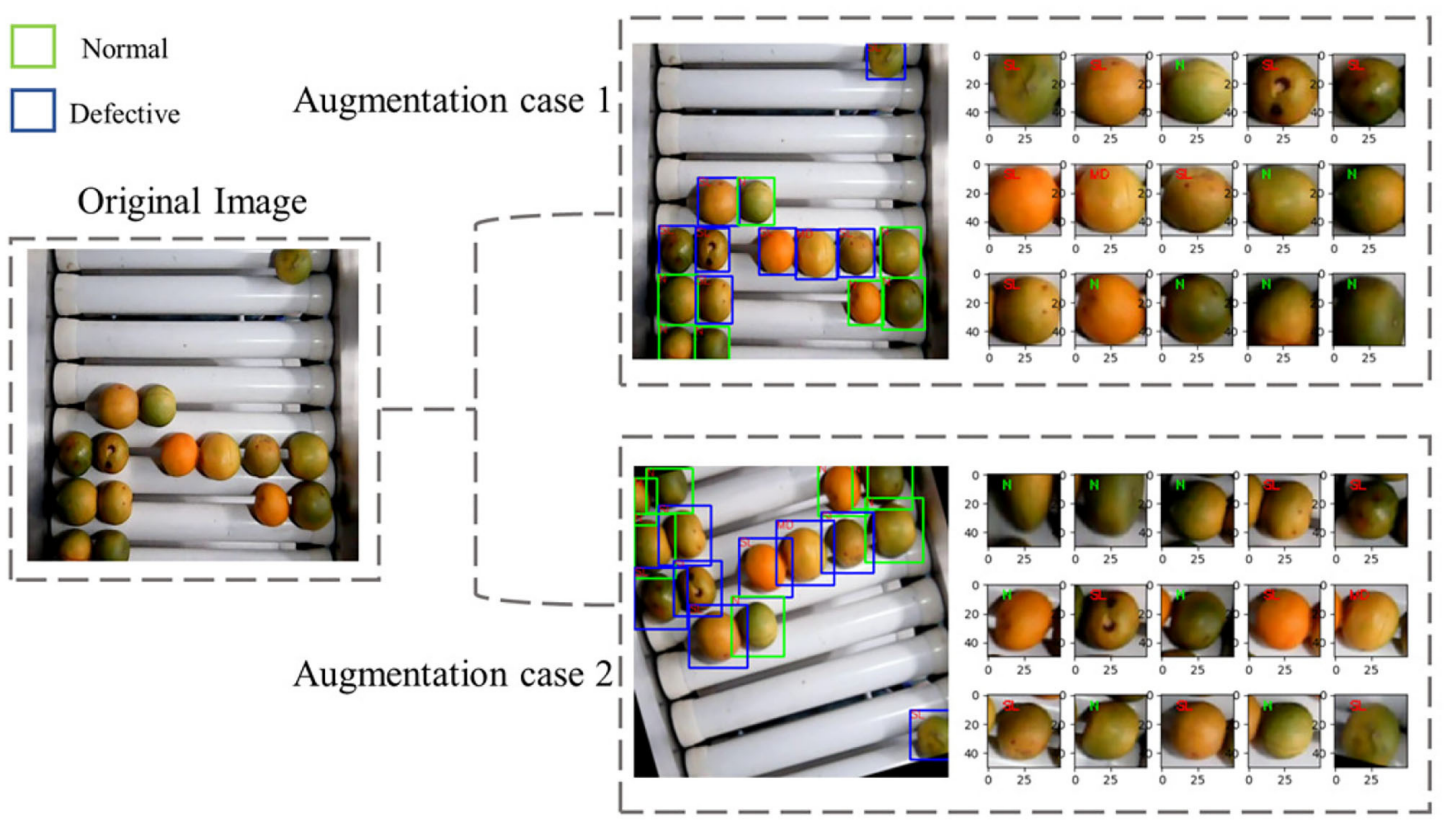
The example of augmented images and labels in network model training.

Multiple image augmentation methods were applied during the training, including scaling (0.8–1.2), flip (in horizontal and vertical direction), rotation (±20°), and adjustment of saturation (0.8–1.2) and brightness (0.8–1.2) in HSV color space, as shown in Figure 5. Adam-optimizer was used to train the network, and the batch size was 24 with the training image resolution of 416 × 416. During the training process, we froze the weight within the backbone network and only trained the detection branch. The network was trained with a learning rate of 0.001 for the first 80 epochs and another 40 epochs with a learning rate of 0.0001.

### 2.3. Defective Citrus Tracking

Defective citrus fruits can have both fine and damaged/lesion surfaces over the fruit body. As the conveyor continuously rotated the oranges, the proposed detector alone could capture multiple surfaces of each fruit, thus possibly labeling the same orange differently in different images. To achieve better detection accuracy, a real-time object tracker was therefore proposed to track and record the classification information of each orange on its path within the working space. The vision system could then classify the true categories of each orange based on the historical classification information.

#### 2.3.1. SORT

**Figure 6:**
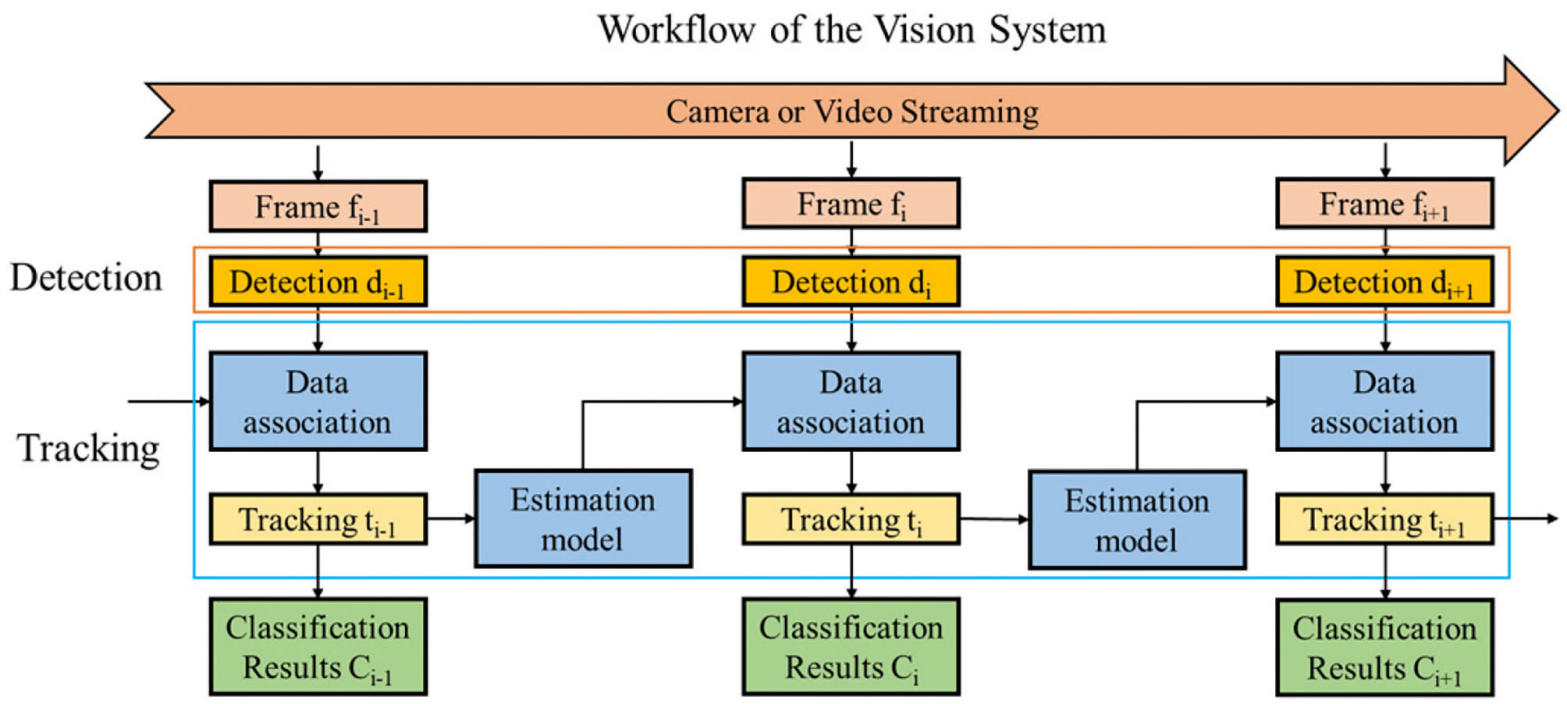
Workflow of the vision system combing a detector and a tracker.

We implemented the SORT algorithm, which is a tracking-by-detection framework-based Multiple Object Tracking (MOT) algorithm (Bewley et al., 2016), as the real-time object tracker for the oranges. SORT has been applied in many vision-based applications, such as autonomous driving (Du et al., 2018), pedestrian tracking (Tang et al., 2016; Wojke et al., 2017), and so on (Chen et al., 2017; Janai et al., 2017; Kosiorek et al., 2018). SORT included two modules: the estimation model and data association, as shown in Figure 6. The estimation model used a linear constant to approximately estimate the motion of the oranges, with the state of each formulated as:

(1) $$\begin{array}{r} x={\left[u,v,s,r,\overset{∙}{u},\overset{∙}{v},ṡ\right]}^{T} \end{array}$$

where *u* and *v* are the horizontal and vertical position of the orange center within the image, and *s* and *r* are the scale and aspect ratio of the bounding box, respectively. If a new detection was matched with an existing tracked orange, the bounding box of the new detected orange was used to update the existing orange's state and predict the bounding box in the next image frame based on the linear Kalman filter model. Data association was solved using the Hungarian algorithm, and the similarity between the predicted bounding box and the new detected bounding box was computed via Intersection-Over-Union (IOU). A minimum threshold was adopted to reject the assignment when the area intersection between the matched bounding boxes was lower than IOU_*min*_.

#### 2.3.2. Classification From Tracking

During the sorting process, the detector detected all the oranges within the working space and temporarily classifies them into Category N, SL, and MD in each image. However, the recognition error would exist when a defective orange presents its sound surface to the camera when rotating. Here, we proposed a new classification strategy that determined the true category of each orange from the tracking process. As the tracker used the detected bounding boxes to track and record the corresponding way-points and classification information of each orange, the vision system recorded a historical list. A logical tree could then be applied to examine the historical list of every orange and identify its true category.

**Figure 7:**
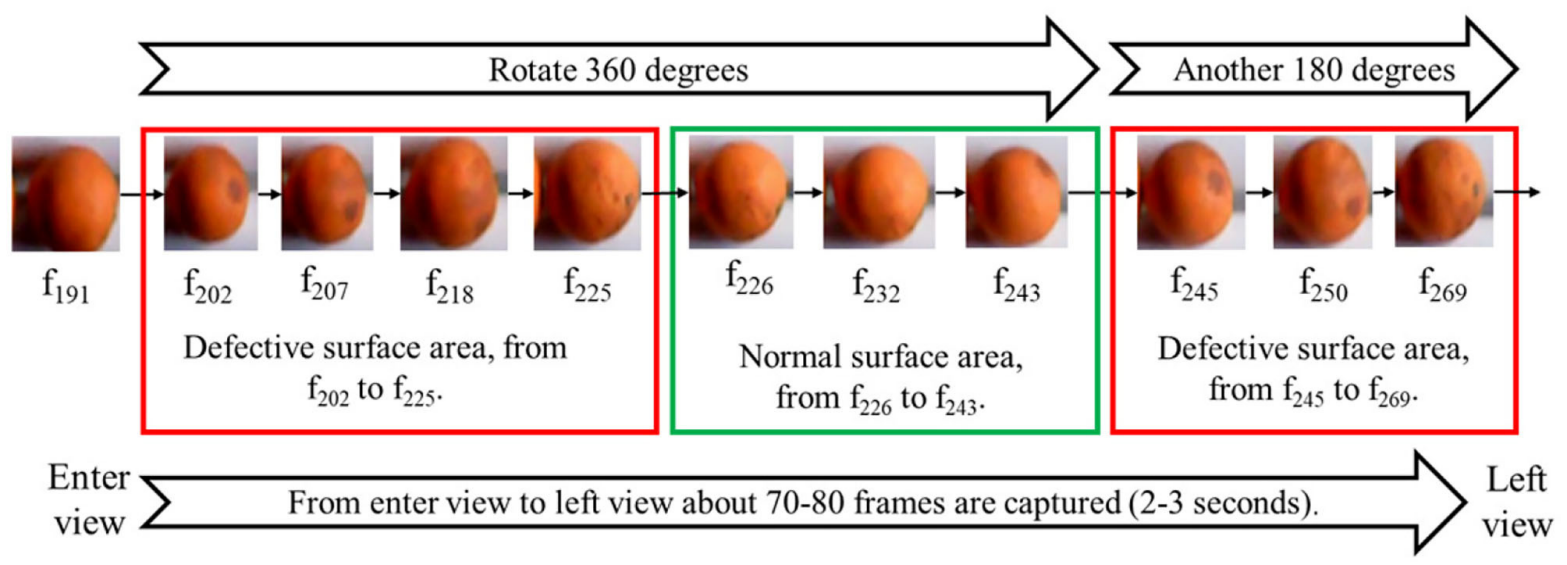
The captured images of a defective orange in 2–3 s, which includes 78 images. The defective part exists in a series of neighboring frames.

As shown in Figure 7, although each orange rotated at a different speed, it rotated roughly 540 degrees when the camera took 70–80 frames in 2–3 s. As a result, if a defect existed on the surface of an orange, it would be captured in a series of neighboring frames. We divided every 8 continuous images as a set of the historical list, and the true category of the orange would be labeled as SL or MD if more than 1 frame in a set was labeled correspondingly. Such a strategy can eliminate some random recognition errors and improve detection accuracy. The classification information would keep updating when the oranges were in the working space, and the oranges would be labeled as N if they were not classified as true SL or MD yet.

### 2.4. Implementation Details

The implemented code of Mobile-Citrus was programmed using the slim library in Tensorflow-1.13, and the model and pre-trained weights of the MobileNet-v2 were from Github publicly code library. The implemented code of SORT was built based on FilterPy library. The overall code of the vision system was built on python 3.5 and performed on windows-10 and Linux ubuntu 16.04. The running speed test was conducted using an NVIDIA-GPU GTX-1660Ti with an Intel-CPU i7-9750 on Linux ubuntu 16.04.

## 3. Results and Discussion

### 3.1. Evaluation Metrics

The performance of the vision system is evaluated from two aspects: the performance of the detector alone and the performance of the combination of the detector and tracker. In the first experiment, the detector alone is evaluated working on a single image without considering continuous tracking during the sorting process. The *F*_1_ score measures the overall performance of detection, which is formulated as follow:

(2) $$\begin{array}{r} F_{1}=\frac{2*recall*accuracy}{recall+accuracy}, \end{array}$$

where recall measures the fraction of true-positive objects that are successfully detected, and accuracy measures the fraction of true-positive objects within the detection.

In the second experiment, the overall performance of the vision system is evaluated using the Multiple Object Tracking Accuracy (MOTA) and Multiple Object Tracking Precision (MOTP). The MOTA is formulated as below:

(3) $$MOTA=1-\frac{\sum_{t}\left(m_{t}+fp_{t}+mme_{t}\right)}{\sum_{t}g_{t}}$$

where *m*_*t*_ and *fp*_*t*_ measure the total number of miss and fault results within detection, respectively, and *mme*_*t*_ measures the mismatched objects within the tracking process. *g*_*t*_ is the ground truth of object tracking at time *t*. The MOTP is formulated as follow:

(4) $$MOTP=\frac{\sum_{i,t}d_{t}^{i}}{\sum_{t}c_{t}}$$

where $d_{t}^{i}$ is the Intersection Over Union (IOU) value between the predicted ground-truth locations and *c*_*t*_ is the number of the correct matched objects, respectively. Higher MOTP and MOTA indicate a better performance of the vision system.

### 3.2. Performance Evaluation

#### 3.2.1. Evaluation of the Detector

**Figure 8:**
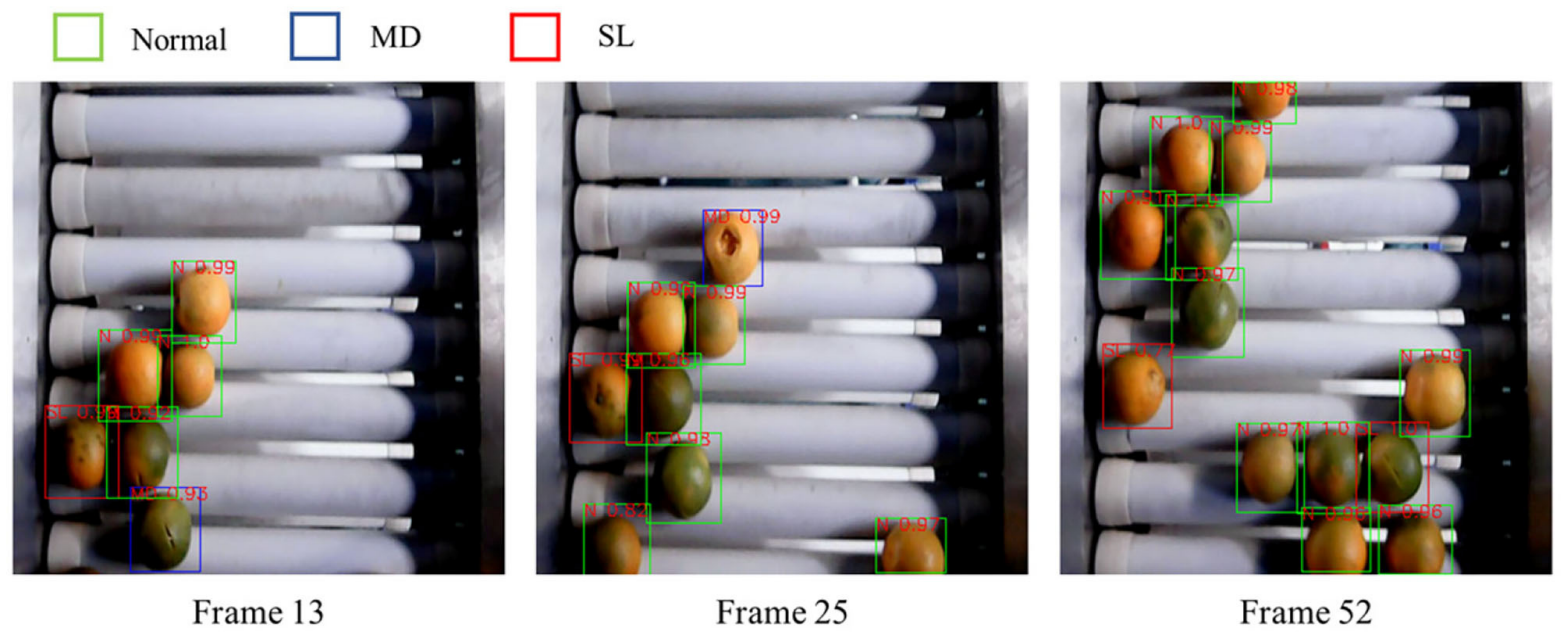
Defective detection results by using the mobile-citrus network. The detected green boxes are normal mandarins while detected red boxes are skin lesion or mechanical damaged mandarins.

We first evaluate the performance of the detector, Mobile-Citrus. A threshold value 0.5 is used to filter unmatched bounding boxes. The experimental results of the detector on defective citrus detection are presented in Table 1 and Figure 8.

**Table 1:** Performance evaluation of the detector alone.

**Categories**	**Accuracy**	**Recall**	**F1-score**
Normal (N)	0.92	0.86	0.883
Defective (SL+MD)	0.85	0.92	0.868
Surface lesion (SL)	0.84	0.94	0.872
Mechanical damaged (MD)	0.86	0.91	0.875
Overall (N+SL+MD)	0.87	0.88	0.871
Overall (no classification)	1.0	0.99	0.99

The overall recall, accuracy, and *F*_1_ score achieved by the detector are 0.87, 0.88, and 0.871, respectively. To further evaluate the model performance in different categories, we separate the classification into Normal and Defective cases, where Defective case includes Category SL and MD. The detector has higher accuracy but lower recall on Normal oranges, while it has higher recall but relatively lower accuracy on Defective ones. This is possibly due to the varietal green to orange colors on the surface of this type of oranges. Since the lesional areas on the defective oranges, which are usually presented in dark and rotten appearances, are similar to the darkly green area on normal oranges, the recognition accuracy can be influenced. As a result, the detector tends to classify Normal oranges into Defective ones in a small amount of cases.

The recall and accuracy of the detector without considering classification error are 0.99 and 1.0, respectively, indicating its capability to detect all the oranges within the working space. It has to be noted that, as shown in Figure 8, even defective oranges have both normal and defective surfaces, which are captured in different images and temporarily classified as Normal and Defective cases respectively. A true Normal orange, however, should present a sound surface to the camera all the way during rotation. Therefore, the detector alone cannot classify the true category of the oranges, and tracking is an indispensable step to track oranges along their paths and identify the true SL and MD ones.

#### 3.2.2. Evaluation of the Combination of the Detector and Tracker

**Figure 9:**
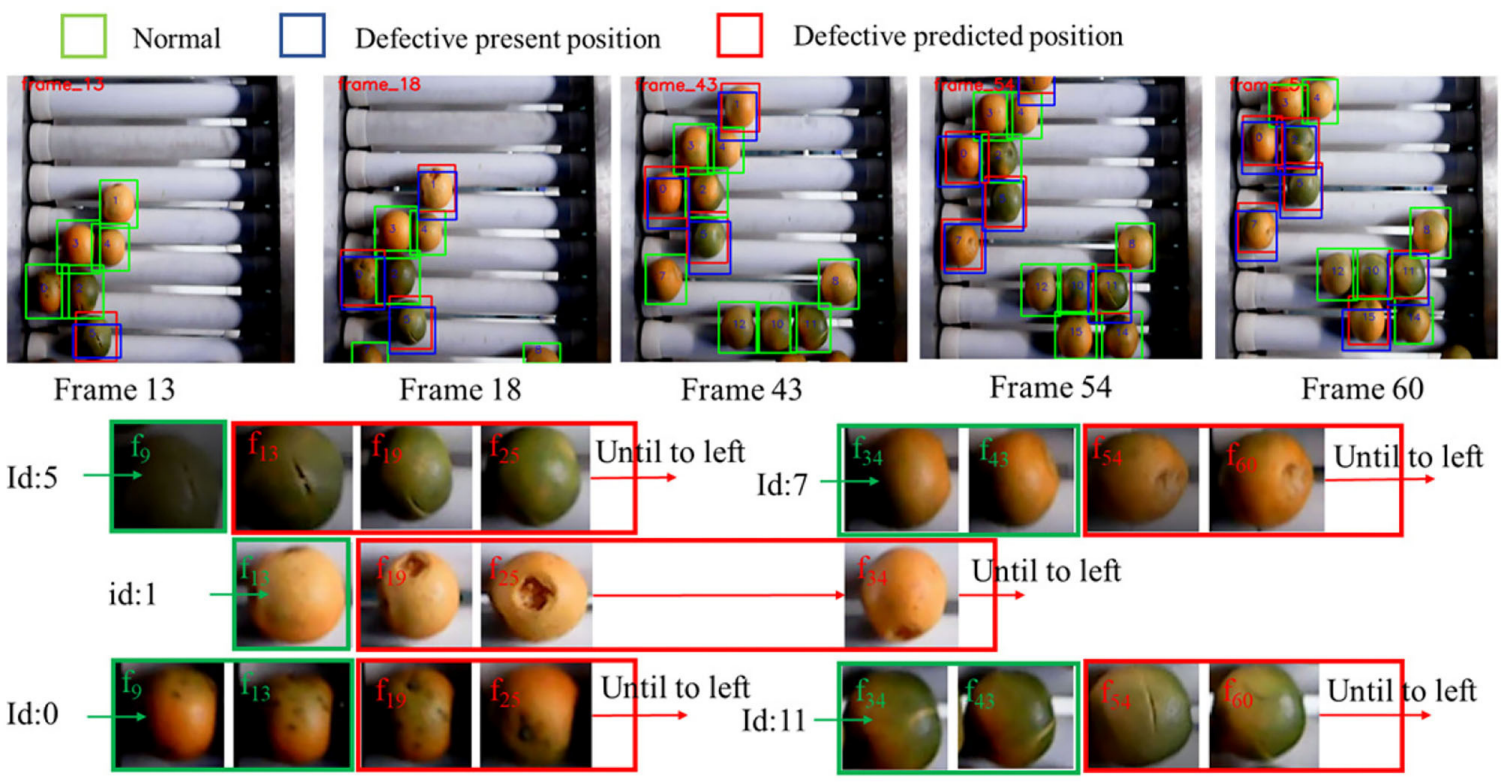
Defective detection and tracking results by using the developed methods. The tracking series of five identified defective mandarins are shown in figure. Green box stands that this mandarin is identified as normal case, while red box stands that this mandarin is identified as defect.

The tracker enables the vision system to memorize the historical classification information and track the location of each orange. The MOTP (also includes m_*t*_ and fp_*t*_, as described in section 3.1) and MOTA are used as metrics to measure the performance of the combination of the detector and tracker. The experiment adopts the recorded tracking list to perform classification and the object tracking dataset performs the evaluation. The results are summarized in Table 2 and an example of the results is shown in Figure 9.

**Table 2:** Performance evaluation of the combination of the detector and tracker.

**Categories**	**MOTA (%)**	**m_***t***_ (%)**	**fp_***t***_ (%)**	**MOTP (%)**
Normal (N)	93.7	3.68	2.53	85.5
Defective (SL+MD)	93.4	2.83	3.76	85.5
Surface lesion (SL)	93.6	2.57	3.84	85.6
Mechanical damaged (MD)	93.4	3.13	3.46	85.4
Overall (N+SL+MD)	93.6	3.5	2.9	85.5

It can be observed that the proposed strategy significantly improves the accuracy of the sorting process. The overall MOTA is 93.6%, and m_*t*_ and fp_*t*_ within MOTA are 3.5 and 2.9%, respectively. The error distribution of the system is different in the cases of Normal and Defective oranges. For the Normal oranges, the system has a larger error in miss classification (m_*t*_ is 3.68%) while is more accurate in false classification (fp_*t*_ is 2.53%). However, for the Defective oranges, the system can identify most of the defective ones (m_*t*_ is 2.83%) but the classification accuracy is relatively lower (fp_*t*_ is 3.76%). These results demonstrate that our vision system can classify the true categories of most of the oranges. However, it also tends to misclassify the normal oranges as Defective ones in a small portion of the cases, possibly also due to the similar appearance between the dark green surface and defective area. The MOTP score of the tracking algorithm is 85.5%, demonstrating a highly precise performance on estimating future locations of the oranges. This also indicates that the velocity of each orange on the conveyor is relatively constant.

#### 3.2.3. Evaluation of the Running Time

In automated citrus sorting, real-time performance is essential as high-speed updating of the new vision information secures the accuracy and success rate. The proposed vision system consists two components, a detector and a tracker, and their average running time are presented in Table 3.

**Table 3:** Average running time of detection and tracking algorithms.

**Number of objects**	**Fraction (%)**	**Detection (ms)**	**Tracking (ms)**	**Total**
<8	15	10	7	17 ms (59 FPS)
8–20	72	11	12	23 ms (43 FPS)
> 20	13	12	22	34 ms (30 FPS)

We count the frequency of the orange number within an image within the dataset and denote it as the fraction in Table 3. As shown in the results, the average running time of the detector is from 10 to 12 ms [increasing processing time is required in Non-Maximum Suppressing (NMS) algorithm], which is equal to 83–100 Frame Per Seconds (FPS) and indicates good real-time performance. The average running time of the tracker depends on the number of oranges requiring processing. Considering that there are usually 8–20 oranges in each image captured by the camera, the average processing time of the tracker is 12 ms. Overall, the total processing time of each input image when combining the detector and tracker is 23 ms, which is equal to 43 FPS and shows good potential to update the vision information in real-time.

### 3.3. Discussion

The classification accuracy obtained through the combination of the detector and tracker (93.6%) is higher than the results using a similar method (Fan et al., 2020), which yields 90.9% for the Defective fruits and only 83.3% for the Normal ones. A major reason is that the classification-by-tracking strategy proposed identifies a fruit as a true Defective case only if it is temporarily classified as Defective in more than one image in the neighboring 8 ones, resulting in a decrease in random recognition errors. Moreover, instead of performing on-line detection on one fruit at a time, our proposed system can perform detection and tracking on multiple objects simultaneously, leading to significantly improved performance and efficiency. Compared to the results obtained through other methods, such as MSI (Qin et al., 2012), the detection accuracy is similar. Although MSI has its merits in the detection of early decay in the fruits, the proposed vision system has higher detection speed and significantly lower costs. Moreover, the images are captured and analyzed through a conveyor in this study, which complicates the working conditions due to mechanical vibrations, fruit movement, and the increased number of fruits.

The experimental results show that the false classification rate of the vision system on normal and defective oranges are 2.53 and 3.76%, respectively, while the miss detection rate on normal and defective oranges are 3.68 and 2.83%. False classification rate and miss detection rate respectively measure the faction of false-classified oranges and miss-detected oranges in the detection process. When considering only the classification on the normal and defective oranges, the number of false-classified normal oranges should equal the number of miss-detected detective ones and vice versa. The above experimental results indicate that our system has a relatively high recall rate on the detection of defective oranges but the accuracy of the classification is lower. This is due to the similar appearance between the defective part and dark green area on the normal oranges, and a better performance can be expected when it works on another type of oranges with a uniform skin color at the mature stage.

## 4. Conclusions

The focus of this study is to develop a novel vision system to realize fast on-line citrus sorting. A CNN-based detector is adopted to temporarily detect the defective oranges in each image, and a SORT algorithm-based tracker is used to identify the true categories of the oranges from the tracked historical information. The combination of the detector and tracker can detect and track multiple fruits simultaneously, yielding a high overall detection accuracy of 93.66%. The results of this study demonstrate three advantages of the vision system: (1) it can perform detection, tracking, and motion estimation of the defective oranges in a highly accurate and real-time behavior; (2) the algorithms adopt a deep learning network-based architecture, which largely improves the accuracy and robustness of the system; (3) it does not require any modification on the original processing line, which can facilitate our vision system to be promoted and implemented in a wide range of applications with similar working scenarios. Overall, the developed vision system achieves good accuracy and real-time performance that can meet the demand of packing houses for fast on-line citrus sorting.

## Data Availability Statement

The raw data supporting the conclusions of this article will be made available by the authors, without undue reservation.

## Author Contributions

YC conceptualized the experiments, collected and analyzed data, and wrote the manuscript. XA set up the platform and collected data. SG collected the data. SL is the project supervisor. HK selected and trained algorithms and wrote the manuscript. All authors contributed to the article and approved the submitted version.

## Conflict of Interest

The authors declare that the research was conducted in the absence of any commercial or financial relationships that could be construed as a potential conflict of interest.
